# Medical Equipment Planning in Ambulatory Surgery Centers: Enhancing Efficiency, Innovation, and Patient Care

**DOI:** 10.7759/cureus.86388

**Published:** 2025-06-19

**Authors:** Bishan Nandy, Meenakshi Jha

**Affiliations:** 1 Hospital Administration, University of Illinois Hospital and Health Sciences System, Chicago, USA; 2 Dentistry, PharmaACE GmbH, Mezzagoring 10, Reilingen, DEU

**Keywords:** ambulatory surgery centers, building information modeling, clinical workflow, equipment lifecycle management, healthcare technology, medical equipment planning, operational efficiency, predictive maintenance, surgical infrastructure, vendor management

## Abstract

Medical equipment planning plays a pivotal role in the success and sustainability of ambulatory surgery centers (ASCs), where streamlined operations and high-quality patient care are essential. This article explores the strategic process of equipment planning, highlighting its critical phases: procurement, design integration, commissioning, and ongoing maintenance. By aligning clinical needs with operational goals, effective planning ensures the selection and placement of technology that enhances workflow, reduces downtime, and complies with regulatory standards. The integration of advanced technologies such as artificial intelligence (AI), building information modeling (BIM), and predictive maintenance tools is also discussed, emphasizing their role in promoting innovation and long-term sustainability. Additionally, the importance of vendor relationships, staff training, and life cycle management is underscored as key drivers of performance and cost-efficiency. Overall, comprehensive equipment planning in ambulatory surgery centers supports improved patient outcomes, operational resilience, and adaptability in an evolving healthcare landscape.

## Introduction and background

Ambulatory surgery centers (ASCs) have emerged as vital components of modern healthcare, offering cost-effective, efficient, and high-quality alternatives to hospital-based outpatient procedures. The proliferation of ASCs is driven by the growing demand for minimally invasive surgeries, advancements in medical technologies, and shifting patient preferences toward more convenient care settings. These centers perform a wide range of procedures, such as orthopedic, ophthalmologic, and gastrointestinal surgeries in an environment that prioritizes streamlined workflows and shorter patient recovery times, contributing significantly to healthcare system efficiency and patient satisfaction [1,2].

As of recent data, there are more than 6,100 Medicare-certified ASCs in the United States, performing over half of all outpatient surgical procedures. In 2021 alone, Medicare paid ASCs more than $5 billion in facility fees, highlighting their significant and expanding role in ambulatory healthcare delivery [3-5]. This broad impact means that inefficiencies in key areas, especially medical equipment planning, can significantly affect operational effectiveness, patient outcomes, and overall healthcare costs.

Unlike hospitals, ASCs operate with smaller footprints, focused service lines, and tighter operational margins, requiring streamlined, highly coordinated planning. Medical equipment planning refers to the structured process of identifying, selecting, integrating, and maintaining the tools and devices needed for patient care within a healthcare facility. In the ASC context, this involves aligning equipment with procedural needs, available space, infrastructure, and long-term goals. The impact of poor equipment planning is not hypothetical. In one reported case, a new ASC experienced a six-month delay in opening because electrical specifications for imaging equipment were miscalculated, requiring costly redesign and reinstallation. In another example, underestimating sterilization equipment needs led to surgical backlogs, directly affecting patient throughput and revenue. These examples highlight how technical oversights can cascade into operational and financial setbacks.

Within this rapidly evolving landscape, medical equipment planning is a foundational element that directly influences the operational success and clinical outcomes of ASCs. Strategic planning ensures that equipment selections align with clinical goals, workflow needs, and future scalability. Well-integrated medical equipment enhances operational efficiency by minimizing delays, supporting clinician productivity, and reducing maintenance disruptions. Moreover, incorporating innovative technologies can drive clinical advancements and differentiate ASCs in competitive markets. However, inadequate equipment planning can compromise patient safety and expose facilities to legal liability. Failure to adhere to regulatory standards, ensure infection control, or prevent equipment misuse may not only jeopardize care quality but also result in serious litigation and reputational damage [6,7]. At the same time, equipment planning must emphasize safety by adhering to regulatory standards, promoting infection control, and reducing risks associated with equipment misuse or failure [8-10].

Commissioning, which refers to the delivery, installation, and testing of equipment, and predictive maintenance, which uses data to forecast and prevent failures, are often overlooked in fragmented planning models. These processes are essential for long-term safety and performance. However, current literature tends to focus on isolated elements such as procurement or compliance, offering limited guidance on comprehensive planning tailored specifically to ASCs. Unlike hospital-based settings, ASCs must operate with smaller footprints, streamlined staff structures, and high turnover, requiring more agile, space-efficient, and cost-effective equipment strategies to support rapid, outpatient-focused care delivery. For clarity, building information modeling (BIM) refers to a digital representation of physical and functional characteristics of a facility, used to optimize infrastructure planning and coordination. Predictive maintenance involves real-time monitoring and analytics to forecast equipment failures before they occur, reducing downtime and service disruptions.

This article aims to address this gap by providing a practical, life cycle-based framework for medical equipment planning, from strategic visioning and procurement to commissioning, maintenance, and technological innovation.

This article provides a comprehensive overview of medical equipment planning in ASCs, emphasizing its role in enhancing efficiency, fostering innovation, and ensuring patient safety. It begins by outlining the procurement process, including vendor selection, budgeting, and evaluation criteria such as clinical relevance, cost-effectiveness, and regulatory compliance [11,12]. Next, it explores the integration of equipment within facility design, focusing on layout optimization, infrastructure compatibility, and regulatory considerations [13,14]. The article then examines commissioning processes, staff training, and ongoing maintenance, highlighting best practices for life cycle management and performance optimization [15]. Finally, it discusses emerging trends in predictive maintenance and the application of digital tools such as BIM and Internet of Things (IoT) in Industry 4.0 environments [16,17]. Together, these insights underscore the critical role of thoughtful equipment planning in supporting the mission and sustainability of ASCs.

## Review

Visioning: Setting the strategic direction

Visioning is the foundational phase in medical equipment planning that establishes the strategic direction and long-term objectives of an ambulatory surgery center (ASC). It involves defining the center’s mission, clinical priorities, and operational goals in alignment with both current needs and future aspirations. The primary objective of this phase is to ensure that all subsequent planning decisions, particularly regarding equipment selection and integration, are purpose-driven, cost-effective, and adaptable to evolving healthcare demands.

This process requires active collaboration among key stakeholders, including surgeons, administrative leaders, and financial planners. Surgeons bring critical insight into clinical workflows, procedural requirements, and emerging technologies relevant to their specialties. Administrative teams contribute knowledge of regulatory compliance, facility operations, and staffing needs, while financial planners assess budget constraints, funding options, and long-term return on investment [1,5]. By engaging all stakeholders early, visioning becomes a shared exercise that fosters organizational buy-in and aligns technical planning with institutional strategy.

Strategic alignment with clinical goals and the demographics of the patient population is essential. Equipment planning must reflect procedural volumes, acuity levels, and community health needs to ensure the ASC remains responsive and competitive. For example, a center specializing in orthopedic or ophthalmologic surgeries must prioritize precision imaging and minimally invasive surgical tools tailored to those services [4].

Importantly, visioning must also accommodate future growth and scalability. This includes anticipating expansions in service lines, increases in patient volume, and integration of advanced technologies. A forward-thinking vision supports not only current functionality but also positions the ASC to adapt to new clinical practices and innovations. Long-term success depends on the ability to evolve without costly overhauls, making scalability a central tenet of strategic visioning [6,9].

Equipment list development: Defining requirements

Developing a comprehensive and accurate equipment list is a critical phase in medical equipment planning for ASCs. This process begins with close collaboration between planners and clinical staff, including surgeons, nurses, and technicians, to ensure that the specific needs of each specialty and procedure are identified and prioritized. Clinical input is essential to ensure that selected equipment aligns with operational workflows, enhances safety, and supports high-quality patient care [8,11].

The equipment list typically spans several key categories, each of which is vital to ASC operations. Surgical equipment includes operating tables, electrosurgical units, and specialized instruments tailored to specific procedures. Diagnostic equipment may encompass ultrasound machines or endoscopic systems, while monitoring equipment involves patient vital sign monitors, anesthesia machines, and recovery area monitors. Sterilization equipment, such as autoclaves and ultrasonic cleaners, is essential to maintain infection control standards and support efficient instrument turnaround [13].

Importantly, the equipment list must be developed with both present requirements and future expansion in mind. As ASCs grow or diversify their procedural offerings, their equipment needs evolve. Planning with scalability allows for smoother transitions when integrating new technologies or increasing procedure volume, preventing unnecessary capital expenditures or operational disruptions [16]. In some cases, it may be practical to develop the equipment plan in phases, prioritizing essential systems initially while deferring non-urgent or specialty equipment to later stages based on evolving needs and budgetary considerations.

Thorough documentation is essential throughout this phase. Each item should be specified with its clinical justification, technical requirements, estimated cost, space and utility needs, and integration considerations. This documentation ensures transparency, which is a key factor, supports budgeting and procurement, and facilitates future audits or upgrades. Moreover, maintaining a well-documented equipment plan helps align all stakeholders around shared goals and timelines, ultimately contributing to a well-organized and adaptable ASC environment [15,18].

Design phase: Integrating equipment with facility layout

The design phase of medical equipment planning in ASCs is critical to ensuring that clinical functionality, efficiency, and safety are embedded in the facility from the outset. This phase demands close collaboration between architects, designers, and clinical stakeholders to translate medical needs into spatial and infrastructural realities. Integrating equipment with the facility layout begins with joint planning sessions, where the insights of clinicians guide the architectural and engineering teams in optimizing clinical workflows and ensuring that the design meets procedural and operational demands [13,19].

Optimizing workflow and accessibility is a cornerstone of effective layout design. Patient flow, from admission through surgery to recovery, must be streamlined to minimize delays and reduce stress for both patients and staff. Space adjacency, clear wayfinding, and ergonomic positioning of equipment play a major role in supporting this goal. Technologies such as process mining and simulation modeling are increasingly used to evaluate layout configurations and predict patient flow patterns to enhance design outcomes [20,21].

Regulatory compliance is another central concern during this phase. The layout must meet standards related to safety, accessibility (e.g., Americans with Disabilities Act (ADA) requirements), and infection control. Design teams must ensure that sterilization areas are adequately isolated, handwashing stations are conveniently placed, and clean and dirty workflows are separated to prevent cross-contamination. Incorporating occupational health and safety standards early in the design also reduces future operational risks [14,22].

Infrastructure integration is equally essential. The layout must accommodate critical systems such as power supply, heating, ventilation, and air conditioning (HVAC), and storage. Equipment such as imaging machines and sterilizers may require dedicated power loads, ventilation, and temperature control. The use of building information modeling (BIM) technologies can support this by aligning mechanical, electrical, and plumbing systems with equipment specifications, thereby preventing costly retrofits or utility mismatches [23,24].

By aligning spatial design with clinical and operational requirements, this phase ensures that ASCs are both functionally efficient and compliant with safety and performance standards, setting the stage for seamless commissioning and operation.

Procurement: Acquiring the right tools

The procurement phase in medical equipment planning for ambulatory surgery centers (ASCs) is a strategically important process that ensures clinical environments are equipped with the right tools to support high-quality, efficient, and safe care. A well-structured procurement strategy involves vendor selection, contracting, and budgeting, each stage critical to aligning organizational goals with operational capabilities.

Overview of the Procurement Process

Vendor selection begins with identifying suppliers that align with the ASC’s strategic, clinical, and financial goals. Criteria such as quality standards, delivery reliability, support services, and reputation guide the selection process [11,25]. Budgeting is the foundational financial planning stage and typically precedes contracting. It involves estimating upfront costs, ongoing maintenance, and future upgrade expenses. Establishing a clear budget ensures fiscal responsibility, secures financial authorization, and allows for efficient resource allocation [26].

Following this, contracting formalizes the procurement relationship, detailing equipment specifications, delivery timelines, warranty terms, training provisions, and post-installation support [12].

Key Factors in Equipment Selection

Clinical relevance is the foundational criterion: equipment must directly support clinical tasks and meet procedural requirements. Input from surgeons, nurses, and other clinical staff is essential in defining what is functionally necessary for optimal patient outcomes [8].

Quality and reliability are paramount in healthcare, where equipment failure can compromise patient safety. Equipment must have a proven track record of performance, supported by data and vendor credibility [11,27].

System compatibility and integration ensure that new equipment integrates seamlessly with existing IT systems, electronic health records, and other medical devices, reducing workflow disruptions and enabling interoperability [28].

Cost-effectiveness involves evaluating both initial purchase costs and long-term operational expenses, such as maintenance, consumables, and energy use. Life cycle cost analysis is a valuable tool for assessing total ownership costs [29].

Regulatory compliance is a legal and ethical requirement. Equipment must meet national and international standards for safety, hygiene, and performance to be eligible for clinical use [30,31].

Scalability is crucial for future-proofing procurement decisions. Equipment should accommodate future volume increases, technological advancements, and clinical service expansions without requiring complete replacement [32].

Vendor support and training determine how well equipment is adopted and maintained. Vendors should offer comprehensive onboarding, maintenance training, and ongoing technical support to minimize downtime and user error [33].

Advanced technologies and innovation can create significant competitive advantages. Equipment that leverages AI, automation, or remote connectivity enhances precision, reduces manual workload, and improves patient outcomes.

Importance of Vendor Relationships and Delivery Planning

Strong vendor relationships are integral to successful procurement. Long-term partnerships foster trust, improve communication, and often result in better pricing, customization options, and priority service. Vendors that understand the unique needs of ASCs can proactively suggest updates and innovations, aligning procurement with evolving clinical demands [34,35].

Effective delivery planning minimizes disruptions to construction or operations. Coordinating logistics such as storage, site access, installation timelines, and preoperational testing ensures that equipment is delivered and installed efficiently and in good condition [36,37].

Commissioning: Delivery, installation, testing, and training

Commissioning is a critical stage in the medical equipment planning process, serving as the bridge between procurement and operational readiness in ambulatory surgery centers (ASCs). This phase ensures that equipment is delivered, installed, tested, and integrated with the facility layout and that staff are trained for safe and effective use.

Logistics of Equipment Delivery and Storage

Careful coordination is required to manage the logistics of equipment delivery and interim storage. Equipment must arrive on schedule and in optimal condition to avoid delays in the facility’s operational timeline. Appropriate storage solutions should be identified in advance to protect high-value items from damage and environmental exposure until installation [36,37].

Installation and Facility Integration

The installation process must align with the facility layout and clinical workflows, integrating medical devices into their designated spaces while ensuring accessibility and safety. This step requires close collaboration between facility managers, biomedical engineers, and vendor representatives. Integration also involves verifying that electrical, HVAC, and data infrastructure are appropriately configured to support the equipment’s functional needs [38].

Testing for Functionality and Safety Standards

Before entering clinical use, equipment undergoes rigorous testing to verify that it meets technical specifications and regulatory safety standards. Testing procedures include power-up verification, calibration, and simulation of use scenarios to detect defects or operational inconsistencies. This final validation step ensures compliance with design criteria and industry regulations [39].

Staff Training for Safe and Efficient Use

Training clinical and technical staff is essential to ensure proper equipment handling, maintenance, and troubleshooting. Vendor-led sessions should cover device operation, safety protocols, and emergency procedures, equipping staff to use new technologies confidently and correctly [40,41].

Commissioning, when executed effectively, supports a seamless transition into operational readiness, minimizing risks and maximizing efficiency from day one.

Ongoing maintenance and life cycle management

Ongoing maintenance and life cycle management are essential for preserving the value, functionality, and safety of medical equipment in ambulatory surgery centers (ASCs). A structured maintenance approach ensures equipment remains operational, compliant, and aligned with evolving clinical needs while minimizing disruptions and long-term costs.

Establishing Maintenance Protocols and Service Schedules

Developing clear maintenance protocols and service schedules is foundational to sustaining equipment performance. These should include routine inspections, preventive maintenance tasks, and both scheduled and unscheduled repairs. Integration of digital tools such as building information modeling (BIM) can enhance maintenance workflows by providing centralized access to equipment data, service histories, and scheduling tools [42]. Predictive maintenance techniques, using data analytics and machine learning, further improve efficiency by forecasting potential failures and enabling timely interventions [43].

Planning for Upgrades and Eventual Replacements

Proactive planning for upgrades and replacements ensures that ASCs remain current with technological advancements and regulatory standards. Midlife upgrades of key equipment, rather than complete replacements, can extend asset lifespans while offering cost-effective enhancements in performance [44]. Life cycle management plans should account for component wear, obsolescence, and future scalability, helping organizations avoid sudden capital expenditures and service disruptions [45].

Ensuring Long-Term Equipment Sustainability and Performance

Sustainability in equipment life cycle management involves a balanced focus on environmental, operational, and economic factors. Condition monitoring and refurbishment planning can support optimal performance over time while minimizing energy and material consumption [46]. For critical systems, such as HVAC or mechanical, electrical, and plumbing (MEP) components, probabilistic modeling supports accurate forecasting of service life and guides long-term resource planning [47]. Incorporating asset management principles, especially in utility systems, further strengthens operational reliability and supports long-term institutional goals [48].

By adopting a proactive, data-informed approach to equipment life cycle management, ASCs can maintain high levels of clinical efficiency, ensure patient safety, and manage costs across the equipment’s lifespan.

Table 1 summarizes the life cycle phases of medical equipment planning in ambulatory surgery centers.

**Table 1:** Life cycle phases of medical equipment planning in ASCs ASCs: ambulatory surgery centers

Phase	Key focus
Visioning	Set strategic direction aligned with clinical and demographic goals
Equipment list development	Define equipment needs in collaboration with clinical staff
Design phase	Integrate equipment with facility layout and regulatory compliance
Procurement	Select and acquire equipment based on quality, cost, and compatibility
Commissioning	Deliver, install, test, and train for operational readiness
Ongoing maintenance	Maintain, upgrade, and sustain equipment performance over time

## Conclusions

Effective medical equipment planning is a strategic pillar for the success of ASCs. Each phase, from visioning and equipment specification to procurement, commissioning, and maintenance, plays a critical role in ensuring clinical efficiency, operational sustainability, and patient safety. When guided by clinical priorities and implemented through thoughtful facility integration and careful procurement, equipment planning lays the groundwork for scalable, high-performing surgical environments. Technology-driven and forward-thinking planning are especially vital for future-proofing ASCs. Tools such as building information modeling (BIM), predictive maintenance systems, and digital life cycle management frameworks allow for smarter, data-driven decisions that adapt to evolving clinical demands and regulatory standards. Equally important is the adoption of practical implementation strategies through adherence to national accreditation standards (e.g., Accreditation Association for Ambulatory Health Care (AAAHC) and Joint Commission) and the use of structured planning toolkits that guide equipment selection, compliance documentation, and integration workflows. Policy frameworks that emphasize risk management, safety compliance, and value-based procurement further support successful execution. Ultimately, a well-conceived and technology-enabled equipment strategy not only streamlines operations but also elevates the quality of patient care. ASCs that embrace robust planning and innovation are best positioned to deliver safe, efficient, and future-ready surgical services.
